# Supplementary material for: Mentorship to strengthen health system leadership: A case study of the Walungu rural health zone in the eastern Democratic Republic of Congo
Source: PLOS Glob Public Health. 2024 Dec 12;4(12):e0003354. doi: 10.1371/journal.pgph.0003354 (PMC11637333; doi:10.1371/journal.pgph.0003354)
Supplement: S1 File — (PDF) [file pgph.0003354.s001.pdf]

Apport du Leadership dans le renforcement de la fonctionnalité et de la performance du système sanitaire : cas de la zone de santé rurale de Walungu, en République Démocratique du Congo

Guide d'Entretiens Approfondis avec les membres de l'ECZ Walungu 2014-2019

Bonjour Madame, Monsieur,

Cette interview est faite pour revenir sur l'expérience de la ZS de Walungu durant les années du programme RIPSEC.

Vous avez été ciblé de par votre fonction, votre expérience et votre rôle dans la ZS durant la période RIPSEC.

Nous allons enregistrer cet entretien, cependant nous vous garantissons le respect total de votre anonymat tout au long de cette étude et même après. L'entretien va durer 30 à 45 minutes.

Avez-vous des questions d'éclaircissement avant de nous donner votre consentement ? Nous sommes disposés à répondre à toutes vos questions. [Répondez clairement aux éventuelles questions. Si le consentement est accordé, procédez à l'entretien. Si non, mettez fin poliment à la conversation et remerciez la personne].

Merci pour le temps que vous nous accordez. A présent je vais allumer l'enregistreur.

A. Questions Générales

1. Pouvez-vous vous présenter et donner le poste que vous occupez actuellement ? (Et cela depuis combien de temps ?)
2. Quel a été votre rôle dans le projet RIPSEC à Walungu ?
3. Pouvez-vous nous décrire en quelques mots ce que les interventions que le projet RIPSEC a menées dans la zone ? *(Sondez : Dans l'appui et l'encadrement de la 1ère ligne des soins au niveau du Centre de Santé ? Au niveau de l'HGR ? Au niveau du BCZ ?)*
4. Quelle est votre appréciation globale de cet encadrement? Quels aspects de cet encadrement, tel qu'il était formulé, auriez-vous orienté autrement si vous en aviez le pouvoir ?

B. Influence des Maisons de Soin Informel sur l'utilisation des services curatifs

1. Dans nos investigations, nous avons noté un nombre assez considérable de maisons de soins informelles (tradipraticiens, maisons de prière de guérison) dans la ZS Walungu. Quelle lecture faites-vous de ce constat ?
2. Pensez-vous que la présence de ces maisons a une influence sur l'utilisation des services que la ZS offre à la population ?
3. De 2015 à 2019, nous avons noté une tendance à l'augmentation de l'utilisation des services dans la ZS. A votre avis, qu'est ce qui pourrait expliquer cela ?

4. En outre, nous avons remarqué que cette utilisation accuse une chute brusque en 2018, avant de repartir à la hausse en 2019. Quels événements de 2018 pensez-vous pouvoir expliquer cette particularité ?

C. Apport du programme RIPSEC dans le renforcement du leadership individuel et collectif

1. Qu'entendez-vous par leadership ?
2. Quels aspects du leadership pensez-vous que l'encadrement du programme RIPSEC a abordé ?
   Sondez : *L'encadrement a-t-il abordé :*
   Organisation du temps de travail ?
   Compréhension de votre rôle de responsable ?
   Interactions entre la première et la deuxième ligne des soins ?
   Renforcement du rôle du BCZ dans l'appui des deux échelons de soins ?

D. Effet de l'encadrement RIPSEC sur la performance et la fonctionnalité de la zone

1. Selon votre opinion, l'encadrement du programme RIPSEC a-t-il eu quelque chose à avoir avec les scores de performance et fonctionnalité observés dans la ZS entre 2015 et 2019 ? De quelle manière ?
2. En particulier, quelles actions initiées par l'ECZ sous influence de RIPSEC pensez-vous ont contribué à l'amélioration des scores de la zone ? *(Sondez. Demandez quelques exemples concrets).*
3. Nous avons mentionné comment l'utilisation des services a augmenté dans la ZS durant les années RIPSEC. Pouvez-vous attribuer cette amélioration au programme RIPSEC ? *(Sondez)*
4. Quelles actions initiées par l'ECZ sous influence de RIPSEC pensez-vous ont pu contribuer à l'amélioration de cette utilisation ?

Merci pour votre collaboration. Nous avons terminé notre entretien. Je vais éteindre l'enregistreur ensuite vous pourrez s'il vous plait me fournir les renseignements suivants :

Age (années) :
Sexe :
Fonction actuelle dans la ZS ou hors ZS :
Ancienneté dans la ZS, dans la carrière :
A participé au projet RIPSEC : Oui/Non

Merci.
