# Supplementary material for: Mentorship to strengthen health system leadership: A case study of the Walungu rural health zone in the eastern Democratic Republic of Congo
Source: PLOS Glob Public Health. 2024 Dec 12;4(12):e0003354. doi: 10.1371/journal.pgph.0003354 (PMC11637333; doi:10.1371/journal.pgph.0003354)
Supplement: Attachment — Submitted filename: Reviewer_PLOS Global Public Health.docx [file pgph.0003354.s003.docx]

PLOS Global Public Health

The effect of mentorship as a means of strengthening leadership in the health system at the operational level: a case study of the Walungu rural health zone in the eastern

Democratic Republic of Congo

**Remarques et commentaires**

- Ligne 70 : Quel est le budget de la santé actuel de la RD Congo ? et quel est le taux d’exécution de ce budget de la santé ?
- Ligne 73 : Est-ce que tous les établissements de soins de santé dépendent du paiement direct de soins de santé par les patients pour assurer leur fonctionnement ou seulement les établissements de soins de santé privés car les établissements de soins de santé publics bénéficient de la subvention du gouvernement ?
- Ligne 74-75 : comment expliquer que les contraintes financières qui dissuadent les malades d’utiliser les services de santé contribuent à l’amélioration de la qualité des soins ?
- Ligne 93 : Quel est le profil /Qualification des gestionnaires des zones de santé ? A notre connaissance, le personnel de santé bénéficie régulièrement des formations de la part du ministère de la santé via les différents programmes et directions ainsi que de la part des partenaires techniques et financiers (PTF)
- Ligne 204 : les établissements de soins de santé informels sont des structures de soins privées qui offrent les soins de santé à la population, mais n’ont pas d’autorisation d’ouverture du ministère de la santé, pas seulement les personnes âgées. Pour la plupart, les établissements de soins de santé informels fonctionnent avec les documents d’ouverture octroyés par les communes, les zones de santé ou les ministres de l’environnement.
- Ligne 213 : Quel est le type d’approche mixte quantitative et qualitative que vous avez utilisé, étant donné que les données quantitatives ont été collectées de 2015-2019 et celles qualitatives de novembre – décembre 2022 ?
- Ligne 230 : La zone de santé de Walungu a combien des établissements de soins de santé ? Combien des établissements de soins de santé appartiennent à l’Etat, aux confessions religieuses, aux privés lucratifs formels et aux privés lucratifs informels ? Cela nous aidera à apprécier correctement des effets de mentorat par le développement de leadership des équipes cadres sur l’utilisation des soins de santé
- Ligne 274 : quels sont les composantes du cadre conceptuel de l’étude ? Parce que la partie qualitative ne fait pas allusion à ces composantes alors que l’analyse qualitative est déductive.
- Ligne 346-349 : expliquez clairement l’influence négative des établissements de soins de santé informels sur l’utilisation des services curatifs à l’ère de la couverture sanitaire universelle ? L’influence négative n’est pas confirmée car la différence entre les AP sûres et AP infiltrées ne soit pas statistiquement significatif (p=0,062).
- Ligne 488-489 : L’article affirme que la pauvreté et le manque de connaissances sont des facteurs importants qui incitent la communauté à préférer les l’influence négative informels. Alors que la littérature nous renseigne que le coût de soins dans le secteur privé (formel et informel) est toujours élevé et supérieur à celui du secteur public.

**Remarques générales** : Comment peut-on parler d’une influence négative des établissements de soins de santé informels sur l’utilisation des soins dans une zone de santé qui n’a qu’1 centre de santé dans une aire de santé pendant que le secteur privé occupe tout espace en termes de couverture sanitaire (accessibilité) ?

**Eddy KIETO-ZOLA**

**Reviewer**
