# Supplementary material for: Mentorship to strengthen health system leadership: A case study of the Walungu rural health zone in the eastern Democratic Republic of Congo
Source: PLOS Glob Public Health. 2024 Dec 12;4(12):e0003354. doi: 10.1371/journal.pgph.0003354 (PMC11637333; doi:10.1371/journal.pgph.0003354)
Supplement: Attachment — Submitted filename: Response to Reviewers.docx [file pgph.0003354.s004.docx]

Rosine Nshobole Bigirinama

Ecole Régionale de Santé Publique

Université Catholique de Bukavu

Avenue Michombero N° 02

Bukavu

Democratic Republic of the Congo

[rosinebigirinama@gmail.com](mailto:rosinebigirinama@gmail.com)

+243 998 660 530

Bukavu, August 28^th^, 2024

To Plos Global Public Health Editors-in-Chief

Re: **Point-by-point responses to the reviewers’ comments / Submission ID : PGPH-D-24-01193**

Dear Catherine Kyobutungi and Madhukar Pai,

We are very pleased to submit the revised version of our manuscript entitled ***“The effect of mentorship as a means of strengthening leadership in the health system at the operational level: a case study of the Walungu rural health zone in the eastern Democratic Republic of Congo”*** (Submission ID: PGPH-D-24-01193) by Rosine Bigirinama et al.

We would like first to thank the Editors and Reviewers for their constructive comments. We have made the changes as recommended, by integrating suggested corrections and providing requested clarifications and answers to the questions raised.

We hope that we adequately addressed issues raised, and that the quality of the manuscript is improved.

Below is an itemized list of changes made to the revised version of the manuscript and point-by-point replies to the specific comments made by the Editors and the Reviewers.
Note: Line numbers correspond to those in the version of manuscript with Track Changes enabled.

**ANSWERS TO EDITORS**

***Editor’s point #1****:*

In the ethics statement in the Methods, you have specified that verbal consent was obtained. Please provide additional details regarding how this consent was documented and witnessed, and state whether this was approved by the IRB.

***Answer to Editor’s point #1:***

We thank the Editors for this comment. We have provided additional details regarding the verbal consent to interviews as detailed below:

Line 397 to line 407: *“The research protocol for this study was approved by the ethics committee of the Université Catholique de Bukavu under the reference number UCB/CIES/NC/019/2021. The approval was granted under the title “Support for Strengthening the Management and Leadership Skills of the National Malaria Control Program Management Team”, which included this sub-study as part of a broader research framework. All stages of the study complied with the relevant guidelines and regulations. For the qualitative interviews, verbal informed consent was obtained directly by the researcher conducting the interviews, who is a member of the research team. The verbal consent process was documented by the researcher immediately following each participant's agreement, noting the date and time of consent. This approach was approved by the ethics committee, taking into account the study's context and the sensitivity of the participant’s roles.”*

***Editor’s point #2****:*

In the online submission form, you indicated that the datasets analysed during the current study are available from the corresponding author on reasonable request.

***Answer to Editor’s point #2:***

We thank the Editors for this remark. We will upload the quantitative dataset as supplementary information. However, for the qualitative dataset, we will upload only the interview guide as a supplementary information. Interview transcriptions will not be publicly available since that would breach compliance with confidentiality committed to respondent and approved by our research ethics board.

***Editor’s point #3****:*

Please provide a/amend your detailed Financial Disclosure statement. This is published with the article. It must therefore be completed in full sentences and contain the exact wording you wish to be published.

***Answer to Editor’s point #3:***

We thank the Editors for this point. We did not receive any funding for this study, so here is our statement: *“The authors received no specific funding for this work.”*

We will ensure that this statement is appropriately integrated during the resubmission process.

**ANSWERS TO REVIEWER #1**

We highly appreciate the Reviewer's attention and interest in this paper. Below are responses to the points raised by the Reviewer.

***Reviewer’s point #1****:*

I congratulate the authors of the article on their high-quality work and their sense of integrity in the conclusion. However, I am still thirsty for data on the indicators they claim to have used to measure the impact of RIPSEC's support. Of the 8 performance indicators announced, only the results for the curative service utilization rate were presented and discussed with significance (Table 4). I would suggest also presenting the results of the other indicators individually, and then summarize them as you have done. In addition, you should describe the method used to aggregate these other indicators. Done as proposed, the article can be published.

***Answer to Reviewer’s point #1:***

We thank the Reviewer for his positive feedback and acknowledgment of the integrity of our study. We appreciate the Reviewer's interest in the analysis used to measure the impact of the RIPSEC program.

In our analysis, we focused on the rate of use of curative services as a key proxy for assessing the population's reliance on IHFs. We estimated that there was no gain in assessing the impact of the influence of IHFs on the other performance indicator because, as mentioned in the manuscript, the rate of use of curative services was the indicator chosen to highlight the influence of IHFs on service utilization, under the assumption that a higher concentration of IHFs in a health area would likely lead to decreased utilization of integrated formal healthcare facilities. However, we also monitored the other performance indicators throughout the study. They are represented in one of the trends shown in Figures 3 (Curative Trend). These indicators were also aggregated to assess overall performance through benchmarking, as detailed in the Methods section, and presented through the Performance trend in Figure 4.

Moreover, as mentioned in the manuscript, these indicators were collected and aggregated at the Health Area and Health Zone levels by the mentor during the RIPSEC period. We accessed this aggregated data in March 2022 for the purpose of this study.

Finally, we are committed to transparency and have made the databases, including the Excel sheets with aggregation formulas, publicly available alongside the paper. This ensures that all interested parties can fully examine the detailed data and methodologies used.

We believe these details and the information provided in the manuscript adequately address the Reviewer’s concern, and we thank him again for his interest in our study.

**ANSWERS TO REVIEWER #2**

We thank the Reviewer for his overall appreciation of the paper, and for the suggested edits to provide to the text. Here below is a point-by-point answer to them.

***Reviewer’s point #1****:*

Could the authors indicate how and when they proceeded to the integration of data from the two components of the study?

***Answer to Reviewer’s point #1:***

We thank the Reviewer for his observation regarding the integration of data from the two components of the study.

In our study, we employed a sequential mixed-methods approach for exploratory purposes, as described in the "Type and period of study" section. The retrospective quantitative data were accessed for analysis in March 2022. And the qualitative data collection took place in November and December 2022. The integration of the quantitative and qualitative data was conducted during the analysis phase, where findings from the initial quantitative analysis informed the thematic framework of the qualitative interviews. Specifically, the quantitative trends observed (e.g., in curative service utilization) were explored more deeply through targeted qualitative inquiries with key members of the HZMT. This integration allowed us to triangulate the findings and provided a comprehensive understanding of the impact of the RIPSEC program, which is reflected in the thematic analysis described in the manuscript.

We have reformulated the corresponding section of the manuscript to address this insightful Reviewer’s concern and further clarify the analysis process as follow:

Line 233 to line 271: “*We used a sequential mixed-methods approach for exploratory purposes (Table 2), combining quantitative and qualitative components to provide a comprehensive evaluation. Retrospective analysis of key health indicators from 2014 (pre-implementation) through the five-year duration of the RIPSEC project (2015-2019), formed the quantitative component. These data were collected continuously from January 1st, 2015, to December 23rd, 2019, and accessed for analysis on March 23rd, 2022.*

*The qualitative component focused on the program's contribution to leadership and governance development. It was structured around an analysis framework derived from the conceptual framework of the RIPSEC program. The framework centered on three core themes: (1) the influence of IHFs on the utilization of curative services, (2) the impact of the RIPSEC program on strengthening individual and collective leadership within the HZMT, and (3) the effects of RIPSEC mentorship on the overall performance and functionality of the HZ. Data were in-depth interviews that were conducted with key HZMT members, from November 23rd to December 15th, 2022.*

*The integration of the quantitative and qualitative components was achieved during the analysis phase. Quantitative findings informed the development of the qualitative interview guide, while the qualitative data provided deeper insights and helped interpret the trends observed in the quantitative data. Data triangulation from these two components was employed to enhance the robustness of our findings, ensuring that insights gained from one method were corroborated by the other (27-29).”*

***Reviewer’s point #2****:*

Could the authors indicated how they decided to stop the selection of the participants for the qualitative phase. Was the saturation of data attained?

***Answer to Reviewer’s point #2:***

We thank the Reviewer for their insightful comment regarding the selection of participants for the qualitative phase. Initially, we identified 13 HZMT members who had participated in the RIPSEC program and who met our criteria of having served for at least two consecutive years during or overlapping with the program period. Unfortunately, we were only able to successfully contact and obtain informed consent from 7 of these individuals.

Given their roles and involvement in the RIPSEC program, we determined that these 7 participants represented the most relevant and experienced members who could provide comprehensive insights into the program’s impact. Although we did not explicitly set out to achieve data saturation, the depth and consistency of the responses we received led us to believe that additional interviews were unlikely to yield new themes or significant variations in the findings.

We recognize that saturation is a key consideration in qualitative research, and while we did not formally declare saturation, the quality and relevance of the data collected from these 7 participants were sufficient to inform the themes and conclusions drawn in this study.

We appreciate the Reviewer’s input in helping us refine our explanation, and we have revised the manuscript to clarify this point, as follow:

Line 329 to line 336: *“In-depth interviews were conducted with Walungu HZMT members who had served for at least two consecutive years during or overlapping with the RIPSEC program period. Out of 13 eligible HZMT members identified from the RIPSEC archives, 7 (all male) were successfully contacted and provided informed consent to participate. These 7 participants were considered the most relevant due to their significant roles and experiences with the RIPSEC program. Although we aimed to capture a comprehensive range of perspectives, the consistency and depth of the responses led us to conclude that the data collected was sufficient to address the research questions.”*

***Reviewer’s point #3****:*

These are two sections that could be erased to ease the readability: definition of concepts (lines 175-211) and Note (lines 286-300).

***Answer to Reviewer’s point #3:***

We appreciate the Reviewer's suggestion regarding the readability of the manuscript.

While we agree that the "Note" section can be removed to streamline the manuscript, we believe that the "Definition of Concepts" section is crucial for readers who may not be familiar with certain terms specific to the DRC's health system. This section provides essential context that helps ensure the clarity and comprehensibility of the manuscript, especially for an international audience or readers who may not have prior knowledge of these concepts.

Therefore, for example, although “Mentorship” and “Learning and Research Zone” are terms commonly known, in the context of this study, we felt that defining them the way we did would help readers further understand the nature of the support provided by RIPSEC to the HZ managers, as well as how the LRZ functions within the program. This provides necessary context for understanding the study's setting and methodology.

We aim to make the paper accessible to a broader readership, and thus, consider this section necessary. Furthermore, we have slightly revised the lines from 207 to 229 to offer the most clear and detailed information about the concepts used and thank the Reviewer to have pointed out that section. We have deleted the “Note” section as suggested and would be grateful for the Reviewer's understanding of the decision to keep the “Definition of concepts” section.

***Reviewer’s point #4****:*

I would recommend that the interview guide be attached as a supplementary file.

***Answer to Reviewer’s point #4:***

We thank the Reviewer for this suggestion. We have made the interview guide available as a supplementary file.

**REPONSE AU REVIEWER #3**

Nous remercions le Reviewer pour l’attention et l’intérêt accordé à notre travail. Nous apprécions grandement les remarques formulées, qui ont permis d’améliorer la clarté et la solidité de notre travail. Ci-dessous, la réponse à l’observation du Reviewer.

***Point #1 du Reviewer****:*

Ligne 70 : Quel est le budget de la santé actuel de la RD Congo ? et quel est le taux d’exécution de ce budget de la santé ?

***Réponse au point #1 du Reviewer:***

Nous remercions le Reviewer pour cette question pertinente. D’après le rapport 2021 des Comptes Nationaux de la Santé (<https://p4h.world/app/uploads/2024/05/Rapport-sur-les-CNS-2021-RDC-ok.x23411.pdf>), le budget alloué à la fonction santé en 2020 s’élevait à 353 840 902 USD, soit 10,02% du budget national, avec un taux d’exécution de 67,1% la même année, et de 59,6% en 2021. Le taux de décaissement budgétaire pour la santé quant à lui demeure faible, tels que le soulignent et le Rapport d’Analyse du Financement de la Santé 2023 de USAID (<https://www.lhssproject.org/sites/default/files/resource/2023-12/LHSS%20DRC_%20Rapport%20de%20l%27analyse%20du%20financement%20de%20la%20sant%C3%A9%20en%20RDC_%20FR_508C.pdf> ), et le nouveau PNDS 2024-2030. La centralisation excessive et la lourdeur des procédures d’approbation des paiements serait la cause d'importants goulets d'étranglement dans le décaissement et l’exécution du budget en RDC, d’après la Banque Mondiale (<https://risquepays.gouv.cd/statistics/wp-content/uploads/2023/06/Development-Policy-Financing-1-%E2%80%93-Juin-2022.pdf>).

Ainsi, en réponse au commentaire du Reviewer et pour clarifier ce passage, nous l’avons modifié comme suit :

Ligne 69 à ligne 73 *: « En 2021, l'État congolais a alloué 11,4 % de son budget à la santé, mais seulement 59,6 % de cette allocation a été exécutée (5). Avec moins de 15% du budget national généralement alloué à la santé chaque année, et de faibles taux de décaissement et d'exécution, la charge du financement des soins de santé repose en grande partie sur les ménages. Ces derniers contribuent à hauteur de plus de 40 % au budget global de la santé, dont 90 % sous forme de paiements directs.»*

***Point #2 du Reviewer****:*

Ligne 73 : Est-ce que tous les établissements de soins de santé dépendent du paiement direct de soins de santé par les patients pour assurer leur fonctionnement ou seulement les établissements de soins de santé privés car les établissements de soins de santé publics bénéficient de la subvention du gouvernement ?

***Réponse au point #2 du Reviewer:***

Nous remercions le Reviewer pour cet intéressant point de discussion soulevé. S’il est vrai que les établissements de soins de santé publics bénéficient de la subvention du gouvernement de la RDC, cette subvention peine encore à couvrir de manière adéquate les Dépenses Courantes de la Santé (DCS). Selon les Comptes Nationaux de la Santé, en examinant par exemple la période allant de 2016 à 2020, les ménages restent la première source de financement (43% de la DCS en 2020). Leur financement est passé de 658 980 708 à 852 343 618 USD entre 2016 et 2020 soit un accroissement de 29,3%. Les bailleurs occupent la deuxième place (37% de la DCS en 2020). Leur financement est passé de 701 009 122 à 739 671 220 USD entre 2016 et 2020 soit un accroissement de 5,5%. Le Gouvernement vient seulement en troisième position avec 16% de la DCS et son financement est passé de 197 333 273 à 312 434 280 USD soit un accroissement de 58,3%. De plus, nous notons que la rémunération des employés est le premier poste des dépenses de santé du Gouvernement, avec 78% de sa DCS, suivi des produits pharmaceutiques (9%) et des autres dépenses (13%).

Et selon le Rapport d’Évaluation du PNDS 2019-2022 paru en 2023 et cité dans le Rapport d’Analyse du Financement de la Santé de USAID (<https://www.lhssproject.org/sites/default/files/resource/2023-12/LHSS%20DRC_%20Rapport%20de%20l%27analyse%20du%20financement%20de%20la%20sant%C3%A9%20en%20RDC_%20FR_508C.pdf> ), la part des ménages est passé à 44% en 2021, et celle du gouvernement à 15%.

Quant à la Dépense d’Investissement en Santé, qui contribue également au fonctionnement des établissements de soins (notamment la construction/entretien des établissements sanitaires, l’achat d’équipements médicaux,…), celle-ci est revenu régulièrement modeste, ne représentant chaque année que moins de 5% de la Dépense Totale en Santé, les 95% restant étant occupés par la DCS, d’après les Comptes Nationaux de la Santé.

Ainsi, le recours des établissements au paiement direct des soins de santé par les patients pour assurer leur fonctionnement s’observe autant dans les établissement privés que dans ceux publics.

***Point #3 du Reviewer****:*

Comment expliquer que les contraintes financières qui dissuadent les malades d’utiliser les services de santé contribuent à l’amélioration de la qualité des soins ?

***Réponse au point #3 du Reviewer:***

Nous remercions le Reviewer pour cette question. Cependant, nous signalons qu’une erreur s’est probablement glissée dans le processus de traduction de passage auquel le Reviewer fait référence. En effet, ce passage dit mot pour mot ceci : « Ces contraintes financières dissuadent considérablement les malades d'utiliser les services de santé, **ce qui contribue à l'appauvrissement croissant de la population et à la dégradation de son état de santé**. ».

***Point #4 du Reviewer****:*

Quel est le profil /Qualification des gestionnaires des zones de santé ? A notre connaissance, le personnel de santé bénéficie régulièrement des formations de la part du ministère de la santé via les différents programmes et directions ainsi que de la part des partenaires techniques et financiers (PTF)

***Réponse au point #4 du Reviewer:***

Nous remercions le Reviewer d’avoir relevé ce point. Les gestionnaires des ZS, regroupés sous le terme « Équipe-Cadre de la Zone de Santé » (ECZS), sont des diplômés d’institutions supérieures et universités. Leur responsable principal, le MCZ, doit être détenteur au minimum du diplôme de Médecine Générale. Bien que le personnel de santé bénéficie effectivement de formations régulières par le ministère de la santé à travers divers programmes et directions, ainsi que par les PTF, ces formations ne sont pas systématiquement mises en œuvre et suivies de manière uniforme à l'échelle nationale.

La littérature scientifique disponible ne documente pas de manière systématique un programme sectoriel formel consacré au renforcement du leadership des ECZS, malgré l’inscription de cette stratégie dans la Stratégie de Renforcement du Système de Santé (SRSS, 2006). Il n’existe pas de preuves solides, sous forme de curriculum ou de programmes pédagogiques spécifiques, qui montrent une approche formalisée de renforcement du leadership en santé publique pour les ECZS en RDC, du moins telle qu’elle est présentée dans la littérature scientifique.

Par ailleurs, les rares formations dont nous avons relevé trace en ligne ne sont pas régulières et pour la plupart datent de si longtemps qu’entre temps les membres de ECZS qui avaient pu en bénéficier pourraient ne plus être en place dans des ZS, si bien que les ZS sont souvent pilotées par de nouvelles équipes qui n’ont pas eu à bénéficier de ces formations. La formation la plus récente trouvée en ligne est celle implémentée par Management Science for Health de 2015 à 2018 avec l’appui de USAID (<https://msh.org/fr/resources/drc-ihpplus-fact-sheet-on-the-leadership-development-program/> et <https://msh.org/wp-content/uploads/2018/09/ihpplus_ldp_fact_sheet.pdf> ) à l’intention de seulement 78 zones de santé, sur les 519 que compte la RDC.

Cependant nous reconnaissons la pertinence de la remarque du Reviewer et lui en remercions. Ainsi, nous avons modifié le passage en question pour mieux refléter cette réalité tout en reconnaissant les efforts du ministère et des PTF dans ce domaine :

Lignes 92 à ligne 98 : *« Des études antérieures dans l’Est de la RDC ont documenté un déficit notable en leadership parmi les responsables des zones en crise. En effet, bien que le personnel de santé bénéficie régulièrement des formations de la part du ministère de la santé via les différents programmes et directions ainsi que de la part des partenaires externes, la littérature scientifique ne documente pas de programme formel du gouvernement visant au renforcement du leadership managérial des ZS de manière uniforme et systématique sur toute l'étendue de la république ».*

***Point #5 du Reviewer****:*

Les établissements de soins de santé informels sont des structures de soins privées qui offrent les soins de santé à la population, mais n’ont pas d’autorisation d’ouverture du ministère de la santé, pas seulement les personnes âgées. Pour la plupart, les établissements de soins de santé informels fonctionnent avec les documents d’ouverture octroyés par les communes, les zones de santé ou les ministres de l’environnement.

***Réponse au point #5 du Reviewer:***

Nous remercions le Reviewer pour son observation. Toutefois, nous précisons que dans notre définition des établissements de soins de santé informels, il n'est nullement fait mention de structures spécifiques aux personnes âgées. Notre intention était de souligner que ces entités offrent des soins sans disposer de la qualité ou de l'autorisation requises par le Ministère de la Santé Publique. Elles opèrent en dehors des normes officielles, ce qui peut représenter un risque pour la santé des patients, quelle que soit leur tranche d'âge.

En outre, concernant les documents d'ouverture délivrés par des autorités locales telles que les communes, les zones de santé, ou les ministres de l'environnement, nous remercions le Reviewer pour son commentaire. Selon les normes officielles en vigueur en RDC, l'autorisation de fonctionnement des structures de santé doit être délivrée par le Ministère de la Santé Publique. Cela assure que les établissements respectent les standards nationaux en matière de qualité des soins. Les établissements de soins de santé informels, même s'ils disposent de documents d'ouverture provenant d'autres autorités, peuvent ne pas répondre aux exigences de qualité et de sécurité imposées par le Ministère de la Santé, les rendant ainsi non conformes aux règlements en vigueur. En conséquence, ces structures opèrent souvent en marge des normes officielles du secteur de la santé.

***Point #6 du Reviewer****:*

Quel est le type d’approche mixte quantitative et qualitative que vous avez utilisé, étant donné que les données quantitatives ont été collectées de 2015-2019 et celles qualitatives de novembre – décembre 2022 ?

***Réponse au point #6 du Reviewer:***

Nous remercions le Reviewer pour sa remarque pertinente concernant le type d'approche mixte utilisé dans notre étude. Nous avons utilisé une approche mixte séquentielle, telle que nous le décrivons dans notre méthodologie. Ainsi, en réponse à cette observation, nous avons modifié le texte dans notre manuscrit comme suit pour être plus détaillé sur l’approche utilisée :

Ligne 233 à ligne 235 : *« Nous avons utilisé une approche mixte* ***séquentielle*** *à des fins exploratoires (tableau 2), combinant des composantes quantitatives et qualitatives afin de fournir une évaluation complète. »*

Par ailleurs, comme nous l’avons précisé dans la méthodologie toujours, les données quantitatives utilisées dans cette étude n'ont pas été collectées directement par notre équipe. Ces données ont été recueillies de manière routinière par le mentor du programme RIPSEC pour des raisons de suivi de l'implémentation du programme dans la zone de santé. Nous avons accédé à la base de ces données de manière rétrospective pour les besoins de notre étude et l’avons complétée par des entretiens qualitatifs afin d'enrichir la compréhension des tendances observées. Ce décalage temporel a permis d'enrichir l'analyse en combinant les résultats statistiques avec les retours d'expérience des acteurs clés. Par ailleurs, nous signalons qu’en réponse au commentaire d’un deuxième Reviewer qui allait dans le même sens que celui-ci, nous avons remanié les lignes allant de 235 à 271 afin de clarifier plus encore la démarche méthodologique.

***Point #7 du Reviewer****:*

La zone de santé de Walungu a combien des établissements de soins de santé ? Combien des établissements de soins de santé appartiennent à l’Etat, aux confessions religieuses, aux privés lucratifs formels et aux privés lucratifs informels ? Cela nous aidera à apprécier correctement des effets de mentorat par le développement de leadership des équipes cadres sur l’utilisation des soins de santé.

***Réponse au point #7 du Reviewer:***

Nous remercions le Reviewer pour son observation perspicace. Nous reconnaissons que la compréhension de la répartition de ces établissements par propriétaire (État, confessions religieuses, privé formel et privé informel) pourrait fournir un contexte supplémentaire pour évaluer l'impact du mentorat sur le développement du leadership et l'utilisation des soins de santé. Dans notre étude, les données sur l'utilisation des services ont été récoltées à l’échelle de l’Aire de Santé et de la zone de santé plutôt qu'à l’échelle de l'établissement de santé. Bien qu'une analyse comparative entre les différents types d’établissements selon leurs propriétaires n’entrât pas dans le cadre de cette étude, votre observation offre une perspective précieuse qui pourrait certainement être explorée dans des études ultérieures. Dans la recherche actuelle, notre objectif était d'évaluer l'effet plus large du mentorat sur la zone de santé dans son ensemble, ce qui, selon nous, permet de comprendre comment le développement du leadership peut avoir un impact sur les résultats en matière de santé dans des environnements complexes.

***Point #8 du Reviewer****:*

Quels sont les composantes du cadre conceptuel de l’étude ? Parce que la partie qualitative ne fait pas allusion à ces composantes alors que l’analyse qualitative est déductive.

***Réponse au point #8 du Reviewer:***

Nous remercions le Reviewer pour cette observation. Nous apprécions l'attention que le Reviewer porte à l'alignement de l'analyse déductive sur le cadre conceptuel global de l'étude. L'analyse qualitative de notre étude a en effet été guidée par un cadre d'analyse dérivé du cadre conceptuel du programme RIPSEC. Ce cadre était structuré autour de thèmes clés directement liés aux objectifs du programme. Ainsi, reconnaissant l’importance soulevée par le Reviewer de rendre ce lien plus clair dans notre manuscrit, nous avons révisé le manuscrit pour faire explicitement référence à ces composantes du cadre conceptuel dans la section méthodologique dans les lignes 233 à 271, lignes 341 à 343 et lignes 362 à 393.

Nous apprécions grandement la contribution du Reviewer, car elle nous a permis d'améliorer la clarté et la cohérence de notre manuscrit.

***Point #9 du Reviewer****:*

Ligne 346-349 : expliquez clairement l’influence négative des établissements de soins de santé informels sur l’utilisation des services curatifs à l’ère de la couverture sanitaire universelle ? L’influence négative n’est pas confirmée car la différence entre les AP sûres et AP infiltrées ne soit pas statistiquement significatif (p=0,062).

***Réponse au point #9 du Reviewer:***

Nous remercions le Reviewer d'avoir souligné le problème de la signification statistique de l’influence négative des établissements de soins de santé informels sur l’utilisation des services curatifs. Bien que la différence dans le taux d'utilisation des services curatifs entre les AS saines et les AS infiltrées avant le programme RIPSEC ne soit pas statistiquement significative (p=0,062), elle était marginalement proche du seuil de 0,05, suggérant une tendance qui, bien qu'elle ne soit pas entièrement significative au niveau conventionnel, indique quand même une influence négative potentielle des établissements de soins informels. Au cours des années de mentorat, cette influence est devenue statistiquement significative (p=0,007), renforçant l'hypothèse selon laquelle la présence de ces établissements a un impact négatif sur l'utilisation des services curatifs formels. Cette tendance, même si elle n'est pas significative au départ, est cohérente avec l'impact observé pendant la période d'intervention et justifie une étude plus approfondie. Nous reconnaissons que ce point doit être interprété avec nuance et que d'autres études avec des échantillons plus importants ou des méthodologies différentes peuvent être nécessaires pour confirmer pleinement l'influence des établissements de soins informels dans le contexte de la couverture sanitaire universelle.

À la lumière des commentaires du Reviewer, nous avons légèrement révisé un paragraphe de la section discussion afin de mieux refléter cette nuance et de suggérer des pistes pour de futures recherches. Le voici ci-dessous :

Ligne 572-580 : *« Les courbes de tendance pour l'utilisation des services de santé curatifs indiquent une baisse de l'utilisation des services formels dans les zones à forte concentration de ESI, ce qui suggère que les efforts de régulation dans ces zones peuvent être moins efficaces. Cependant, il est important de noter que la différence observée avant l'intervention n'était que marginalement significative (p=0,062), ce qui indique que même s'il y avait une tendance, elle n'était pas fortement confirmée statistiquement. Au cours des années de mentorat, la différence est devenue statistiquement significative, soutenant l'idée que les ESI influencent négativement l'utilisation des services curatifs. D'autres études, éventuellement avec des échantillons plus importants ou des méthodologies différentes, pourraient être nécessaires pour explorer pleinement l'influence des établissements de soins informels sur l’utilisation des services curatifs. »*

***Point #10 du Reviewer****:*

L’article affirme que la pauvreté et le manque de connaissances sont des facteurs importants qui incitent la communauté à préférer les l’influence négative informels. Alors que la littérature nous renseigne que le coût de soins dans le secteur privé (formel et informel) est toujours élevé et supérieur à celui du secteur public.

***Réponse au point #10 du Reviewer:***

Nous apprécions l'observation du Reviewer concernant le coût des soins. En analysant la littérature, il s’avère que la relation entre le coût et le secteur des soins de santé (public ou privé) est complexe et varie en fonction du contexte. Par exemple, dans certains contextes, les soins du secteur public s'avèrent légèrement plus chers que ceux du secteur privé, comme dans le cas de la dentisterie (Tuominen & Eriksson, 2011). En Inde, les soins ambulatoires privés, bien que plus complets, étaient plus chers que les soins publics (Bhatia & Cleland, 2004). De même, une étude réalisée au Mexique a révélé que les coûts étaient moins élevés dans le secteur public que dans le secteur privé, en particulier pour l'assurance maladie privée (Arredondo et al., 1995). À l'inverse, dans certains cas, comme l'insuffisance rénale terminale, les coûts de traitement sont plus élevés dans les établissements publics que dans le secteur privé (Hynes et al., 2012). Une autre étude rapporte que les hôpitaux publics du Sri Lanka ont fait preuve d'une meilleure qualité malgré des dépenses inférieures à celles des établissements privés (Rannan-Eliya et al., 2015).

Ces résultats suggèrent que si les établissements publics ont souvent des coûts directs moins élevés, cela n'est pas universellement applicable dans tous les contextes de soins de santé et n’est pas le seul déterminant pas la préférence des patients. Dans notre manuscrit, nous avons mis en avant que le coût et l'insatisfaction à l'égard du système de santé formel sont des facteurs importants qui incitent la communauté à préférer les établissements informels à Walungu. En outre, rappelant que les établissements de soins formels peuvent être autant des établissements publics que des établissements privés, nous avons clarifié la compréhension de ce concept sur base d’un des commentaires d’un autre Reviewer (lignes 207 à 229).

Nous remercions le Reviewer d’avoir soulevé ce point et avons modifié la section de discussion correspondant à ce point afin d’assurer une compréhension nuancée de la relation coût-préférence, tout en restant concentrés sur les objectifs principaux de notre étude :

Ligne 587 à ligne 602 : *« Bien que la littérature suggère que les coûts peuvent varier considérablement entre les secteurs public et privé, les ESI offrent souvent des coûts directs moins élevés et des options de paiement plus souples, ce qui les rend plus accessibles aux personnes ayant des contraintes financières (8). Au-delà de la question des coûts, l'insatisfaction à l'égard du système de santé officiel, en particulier pour les maladies chroniques qui nécessitent un soutien psychosocial important et des soins centrés sur la personne, est également un facteur important qui pousse les patients vers les ESI (12,35-38). La combinaison de coûts directs immédiats moins élevés, de modalités de paiement souples, d'accessibilité perçue et de soins adaptés à la culture contribue à la préférence de la communauté pour les ESI. »*

***Point #11 du Reviewer****:*

Comment peut-on parler d’une influence négative des établissements de soins de santé informels sur l’utilisation des soins dans une zone de santé qui n’a qu’1 centre de santé dans une aire de santé pendant que le secteur privé occupe tout espace en termes de couverture sanitaire (accessibilité) ?

***Réponse au point #11 du Reviewer:***

Nous apprécions l'observation réfléchie de l'évaluateur. Cependant, nous pensons que même dans les zones de santé où les établissements de santé formels (qui peuvent être autant des établissements du secteur publics que du secteur privés, nous le rappelons) sont fortement représentés, les établissements de soins informels (qui sont aussi des privés, mais offrant des soins non alignés aux normes sanitaires nationales, et ne rapportant pas leurs données sanitaires dans le système national d’information sanitaire) peuvent avoir un impact négatif sur l'utilisation des services de santé formels, pour diverses raisons déjà abordées (coût des soins, flexibilité des modes de payement, soins culturellement adaptés, …). De plus, dans notre manuscrit, nous avons souligné que la couverture des soins de santé formels dans la zone d'étude était déjà très optimale, affichant des taux de couverture supérieurs à 95% durant toute la période d’étude. Malgré ce niveau élevé de couverture en soins de santé formels, la présence des établissements informels représentait toujours un défi important, réduisant l'utilisation des services formels. Ainsi, même avec une couverture de soins formels optimale dans la zone de santé, les établissements informels peuvent avoir un impact négatif sur l'utilisation des services de santé intégrés.
